# Cytokinins are involved in regulation of tomato pericarp thickness and fruit size

**DOI:** 10.1093/hr/uhab041

**Published:** 2022-01-19

**Authors:** Lijun Gan, Mengying Song, Xuechun Wang, Na Yang, Hu Li, Xuexia Liu, Yi Li

**Affiliations:** 1 College of Life Sciences, Nanjing Agricultural University, No. 1 Weigang, Nanjing 210095, China; 2 State Key Laboratory of Crop Genetics and Germplasm Enhancement and the College of Horticulture, Nanjing Agricultural University, No. 1 Weigang, Nanjing 210095, China; 3 Department of Plant Science and Landscape Architecture, University of Connecticut, Storrs, CT 06269, USA

## Abstract

Although cytokinins (CKs) regulate fruit development, no direct genetic evidence supports the role of endogenous CKs in pericarp growth or development or fruit size. Here, we report that the reduction in levels of endogenous active CKs via overexpression of the CK-inactivating enzyme gene *AtCKX2* specifically in fruit tissues resulted in reduced pericarp thickness and smaller fruit size compared with wild-type control fruits. Pericarp thickness and single fruit weight in transgenic plants were significantly reduced. Analysis of paraffin sections showed that the reduced pericarp thickness was due largely to a decreased number of cells, and thus decreased cell division. Transcriptome profiling showed that the expression of cell division- and expansion-related genes was reduced in *AtCKX2*-overexpressing fruits. In addition, the expression of auxin-signaling and gibberellin-biosynthetic genes was repressed, whereas that of gibberellin-inactivating genes was enhanced, in *AtCKX2*-overexpressing fruits. These results demonstrate that endogenous CKs regulate pericarp cell division and subsequently fruit size. They also suggest that CKs interact with auxin and gibberellins in regulating tomato pericarp thickness and fruit size.

## Introduction

Tomato (*Solanum lycopersicum*) is a model plant used to understand the growth and ripening of climacteric fruits. Tomato fruits develop from mature ovaries and undergo development in stages—fruit set, fruit growth, maturation, and ripening. The fruit growth stage is characterized by cell division followed by cell expansion. Both processes play critical roles in the regulation of fruit size; final fruit size is achieved by the coordination of cell division and cell enlargement [1, 2].

Plant hormones such as cytokinins (CKs) may play critical roles in the regulation of tomato fruit growth. Exogenous application of the synthetic CK forchlorfenuron (CPPU) induced fruit set and fruit development [3, 4]. Two peaks of CK accumulation in the ovaries during early tomato fruit development, at anthesis and 5 days after anthesis (DAA) have been reported, suggesting that CKs regulate fruit set and early development [3].

The levels of endogenous CKs in plants are controlled spatially and temporally by the balance between biosynthesis and inactivation [5]. Adenosine phosphate-isopentenyl transferases (IPTs) catalyze the rate-limiting biosynthetic step of CKs, producing isopentenyladenine (iP) nucleotides. Next, the cytochrome P450 monooxygenases CYP735A1 and CYP735A2 convert iP nucleotides to *trans*-zeatin (tZ) nucleotides. Finally, CK nucleotides were converted to active nucleobases by LONELY GUY (LOG) [5, 6].

In contrast, cytokinin oxidase/dehydrogenase (CKX) is responsible primarily for the inactivation of CKs and catalyzes their irreversible degradation [7]. CKX selectively cleaves unsaturated N [6] side chains from tZ, iP, and corresponding ribosides [8]. Overexpression of a *CKX* gene has been used to reduce endogenous active CKs in the whole plant or specific organs and tissues to define the roles of endogenous CK in various biological processes. Werner *et al*. [9] overexpressed *AtCKX2* under the 35S promoter, which resulted in significant reduction of endogenous concentrations of different CKs in transgenic tobacco plants. Compared with wild-type (WT), overexpression of *AtCKX2* in *Arabidopsis* significantly increased CKX activity (~10-fold) and reduced levels of endogenous CK metabolites [10]. Nieminen *et al*. [11] expressed *AtCKX2* in the cambial zone of poplar and silver birch to reduce biologically active CK to determine the role of CKs in cambium development. Li *et al*. [12] used a root-dominant promoter to control the expression of the *AtCKX2* gene to reduce active CKs in poplar roots and observed enhanced root growth and elimination of root sucker development. In addition, active CK levels can be modulated by reversible or irreversible conjugation to a sugar by *O*-glucosyltransferases or *N*-glucosyltransferase, respectively [8].

**Figure 1:**
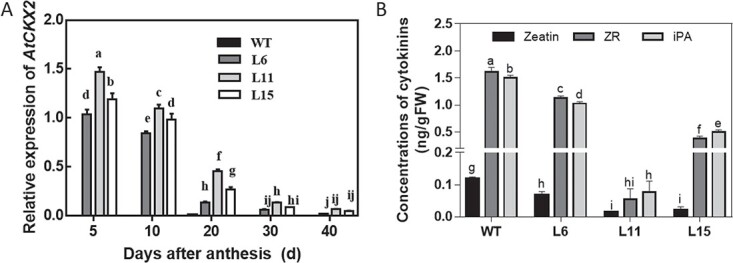
AtCKX2 expression and cytokinin levels in transgenic tomato lines. (**a**) Relative expression of *AtCKX2* in fruits of WT and transgenic lines of tomato. Actin was used as an internal control to normalize data. (**b**) Levels of cytokinins in fruits of WT and transgenic lines. Fruits at 5 days after anthesis were used to measure zeatin, ZR and iPA using UHPLC-TQ-MS/MS. Different letters indicate significant differences at the *P* < .05 level (*n* = 3).

The genetic modification of endogenous CK content has been achieved in tomato via the constitutive expression of *SlIPT* genes [13]. Indeed, the total CK content was up to 12-fold greater in *35S::SlIPT3* lines than in WT plants. However, elevated levels of CKs suppressed tomato growth, and only one transgenic line produced seeds [13]. RNA interference mediated *SlIPT4* gene silencing was used to modify the endogenous CK status in tomato; it accelerated leaf senescence, but caused no significant phenotypic change during fruit growth and development [14]. This lack of effect may be attributable to functional redundancy among *SlIPT* genes or negligible roles of CKs in fruit growth and development. Thus, the roles of CKs in fruit growth remain unclear.

To investigate whether endogenous CKs play roles in tomato fruit growth and development, we used the *Tmf7* gene promoter sequence to control the expression of *AtCKX2* to specifically reduce endogenous CK levels in tomato fruits. The *Tfm7* promoter is fruit-specific; it is active in pericarp, columella, and placental tissues of immature green fruit in tomato [15, 16]. The *AtCKX2* gene has been overexpressed in several species of higher plants to reduce active CK levels [9–12]. Our results demonstrate that endogenous CKs regulate pericarp growth and development, and subsequently fruit size.

## Results

### Endogenous cytokinin levels were reduced in transgenic tomato fruits

To investigate the effects of endogenous CKs on tomato fruit growth and development, three independent transgenic lines (L6, L11, and L15) harboring *Tfm7::AtCKX2* were used. First, we analyzed the expression of *AtCKX2* during fruit development in these three transgenic lines by RT–qPCR. The expression of *AtCKX2* was highest in L11 (Fig. 1a). This expression was high in the early stage of fruit development (5–10 DAA), decreased at 20 DAA, and remained low during the subsequent stages of fruit development (Fig. 1a).

To determine the changes in active CK levels in the transgenic plant lines, using UHPLC-TQ-MS/MS (ultra-high-performance liquid chromatography coupled with triple quadrupole electrospray tandem mass spectrometry), we used tomato fruits at 5 DAA for analyses of zeatin, iP, zeatin riboside (ZR) and isopentenyl adenosine (iPA) levels based on a report of peak CK accumulation at 5 DAA [3]. The level of iP in these fruits was undetectable. The levels of endogenous zeatin, ZR and iPA were reduced in fruits of all three transgenic lines. Consistent with its highest *CKX* gene expression level among the three transgenic lines, L11 had zeatin, ZR and iPA levels that were only 12.5%, 3.1%, and 5.3% of those of the WT control fruits, respectively (Fig. 1b). Thus, *Tfm7-AtCKX2* transgene expression reduced active CK concentrations in fruits.

### Effects of cytokinin deficiency on fruit and seed growth and development

**Figure 2:**
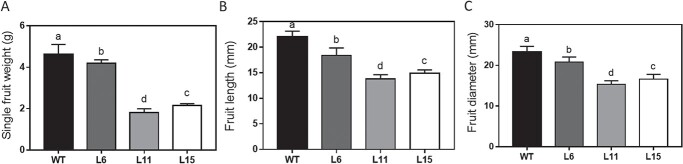
Effects of CK deficiency on weights and sizes of tomato fruits. (**a**) Single fruit weight. (**b**) Fruit length. (**c**) Fruit diameter. Fruits were analyzed at the red stage . Different letters indicate significant differences at the *P* < .05 level (*n* = 15).

To investigate the functions of endogenous CKs in fruit growth, we analyzed the single-fruit weight, diameter, and length of fruits at the red stage. The single-fruit weights of L6, L11, and L15 plants were 87.2%, 32.7%, and 43.3%, respectively, of those of WT fruits (Fig. 2a). L11 fruits were smallest; their lengths and diameters were 62.6% and 65.9%, respectively, of those of WT fruits (Fig. 2b and c). Thus, the reduced endogenous CK levels in transgenic fruits decreased fruit weight and size.

**Figure 3:**
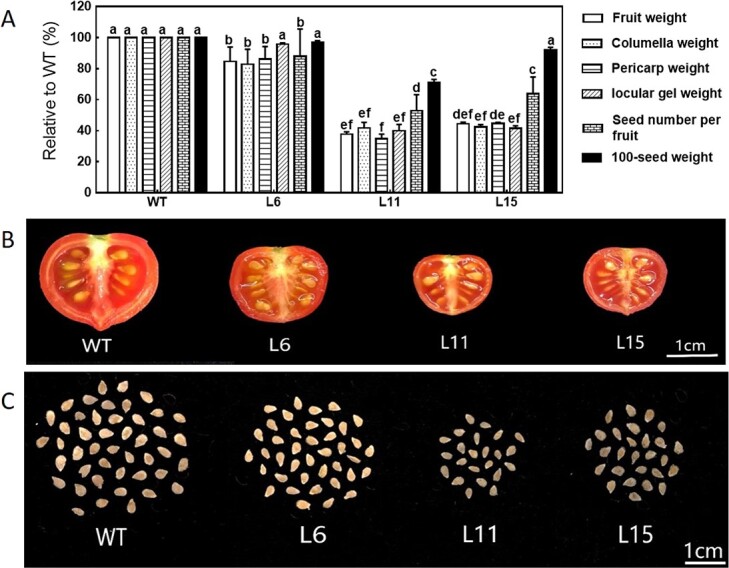
Effects of CK deficiency on various fruit characteristics. (**a**) Changes in fruit characteristics observed in red fruits of transgenic tomato compared with WT. (**b**) Vertical sections of red fruits. Bar = 1 cm; (**c**) Seed size and number in red fruits of WT and transgenic plants. Bar = 1 cm. Different letters indicate significant differences at the *P* < .05 level (*n* = 15).

We next studied the contribution of each fruit part to the decreased fruit weight and size. The weights of the columella, pericarp, and locular gel decreased in proportion to single-fruit weight in L11 and L15 (Fig. 3). In addition, CK deficiency reduced seed number per fruit and 100-seed weight in L11 (Fig. 3), indicating that CKs are important for seed development.

### Effects of cytokinin deficiency on pericarp growth and development

**Figure 4:**
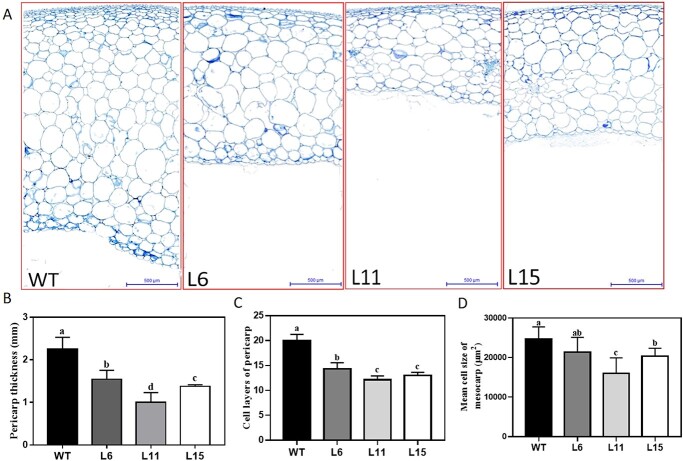
Effects of CK deficiency on pericarp thickness. (**a**) Microscopic cross-sections of pericarp tissues of WT and transgenic fruits. Bar = 500 □m. (**b**) Pericarp thickness. (**c**) Cell layers of pericarp tissues. (**d**) Mean cell size of mesocarp tissues. Fruits at the mature green stage were analyzed. Different letters indicate significant differences at the *P* < .05 level (*n* = 3).

Pericarp tissues of transgenic fruits were significantly thinner than those of WT fruits. The pericarp thickness of WT fruits was ~2.25 mm, whereas those of L6, L11 and L15 fruits were 1.55, 1.00 and 1.38 mm, respectively (Fig. 4a and b). We applied CPPU to L11 fruits and found that exogenous CPPU could restore the phenotype and increase single fruit weight and pericarp thickness in fruits (Supplementary Fig. S1). To determine whether the reduced pericarp thickness was a consequence of a difference in cell size or cell number, paraffin sections of fruits of the WT and the transgenic lines at the mature green stage were analyzed. We counted the cell layers in the pericarp as an indicator of cell division activity. Fewer cell layers were observed in CK-deficient than in WT fruits. L6, L11 and L15 fruits had 14.33, 12.17 and 13.00 cell layers, respectively, compared with 20.00 layers in WT fruits (Fig. 4c). The average cell size in the L6 mesocarp did not differ significantly from that of the WT, but cells of L11 and L15 were smaller on average than WT cells (16 × 10^3^ and 20.4 × 10^3^ vs. 24.7 × 10^3^ μm^2^; Fig. 4d). Thus, the decreased pericarp thickness in CK-deficient fruits was due mainly to reduced cell division activity, but cell size also contributed.

### Transcriptomic analysis of fruits of wild-type and transgenic plants 5 days after anthesis

To gain insight into the molecular mechanism underlying the fruit response to CK deficiency, the transcriptomes of 5-DAA fruits of WT and L11 plants were subjected to sequencing by RNA-Seq. A total of 42.46 Gb of clean data was obtained, of which ≥94.97% had base quality >30. These clean reads were mapped to the tomato genome using Hisat2 software; ~94.19%–94.97% of the clean reads were unique mapped reads (Supplementary Table S1).

Among the total of 35 737 mapped genes in the tomato genome, ~48.27% and 48.84% were expressed [fragments per kilobase of transcript per million fragments mapped (FPKM) >1] in fruits of WT and L11, respectively (Supplementary Fig. S2A). Using the criteria of adjusted *P* < 0.05 and log_2_-fold change (log2FC) ≥1, a total of 496 unigenes, comprising 356 upregulated genes and 141 downregulated genes, were differentially expressed in 5-DAA fruits of L11 compared with WT fruits (Supplementary Fig. S2B).

### Effects of cytokinin deficiency on the expression of cell division- and cell expansion-related genes

Cyclins and cyclin-dependent protein kinases regulate cell division and expansion in tomato plants [17]. *CYCU1;1* (cyclin U1;1, Solyc07g052610) and *CYCU4;1* (Solyc03g093790), members of the cyclin family, were downregulated in L11 fruits (Table 1). Solyc12g098310, which encodes a cyclin-dependent kinase inhibitor (CKI), was also downregulated in L11 fruits (Table 1). In addition, several genes related to cell expansion were differentially expressed in WT and L11 fruits. Three pectinesterase *(PE*/*PME*) genes (Solyc07g064170, Solyc01g091050, and Solyc02g075620), a xyloglucan endotransglucosylase-hydrolase (*XTH5*), a cellulose synthase (Solyc07g005840), two polygalacturonase genes (Solyc12g009420 and Solyc04g008230), an expansin gene (Solyc06g049050), and an extension gene (Solyc01g006390) were up- or downregulated in CK-deficient fruits (Table 1). Among them, two *PE* genes and *XTH5* were markedly upregulated in CK-deficient fruits (log2FC > 3; Table 1).

**Table 1:** Expression levels of cell division- and expansion-related genes in L11 fruits relative to those in WT fruits

**Gene ID**	**Fold change** ^**a**^	**Gene**	**Likely function**
	**(log**_**2**_ **L11/WT)**		
Solyc07g064170	+6.68	Pectinesterase (*PE*/*PME*)	Cell wall stiffening
Solyc01g091050	+5.76	Pectinesterase (*PE*/*PME*)	Cell wall stiffening
Solyc01g081060	+3.18	Xyloglucan endotransglucosylase-hydrolase (*XTH5*)	Cell wall restructuring
Solyc12g009420	+2.81	Polygalacturonase	Cell wall softening
Solyc12g098310	+2.11	Cyclin-dependent kinase inhibitor (*CKI*)	Inhibit cell division
Solyc01g006390	+1.22	Extensin	Cell expansion
Solyc07g052610	−1.10	Cyclin U1;1 (*CYCU1;1*)	Cell division
Solyc04g008230	−1.27	Polygalacturonase	Cell wall softening
Solyc07g005840	−1.25	Cellulose synthase	Cellulose synthesis
Solyc02g075620	−1.29	Pectinesterase	Cell wall stiffening
Solyc03g093790	−1.38	Cyclin U4;1(*CYCU4;1*)	Cell division
Solyc06g049050	−1.39	Expansin 2	Cell expansion

^a^+, increase; −, reduction.

### Effects of cytokinin deficiency on expression of cytokinin-, auxin-, and gibberellin-related genes

The transcript level of zeatin *O*-glucosyltransferase (Solyc04g008330) was reduced significantly (log2FC = −1.43) in fruits of L11 compared with the WT (Table 2). Zeatin *O*-glucosyltransferase converts active CK to reversibly inactivated storage forms and plays an important role in CK homeostasis [18, 19]. In *Arabidopsis*, cytokinin *O*-glucosyltransferase was reportedly upregulated by CKs [20]. The reduced expression level of zeatin *O*-glucosyltransferase in this study is consistent with the decreased endogenous CK levels in transgenic fruits.

**Table 2:** Expression levels of CK-, GA- and auxin-related genes in L11 fruits relative to those in WT fruits

	**Gene ID**	**Fold change** ^**a**^	**Gene**	**Likely function**
		**(log**_**2**_ **L11/WT)**		
CK	Solyc04g008330	−1.43	Zeatin *O*-glucosyltransferase	CK inactivation
Gibberellins	Solyc07g061730	+2.09	*GA2ox5*	GA inactivation
	Solyc06g008870	+1.28	*GID1b2*	GA receptor
	Solyc07g061720	+1.24	*GA2ox4*	GA inactivation
	Solyc03g006880	−1.18	*GA20ox1*	GA biosynthesis
Auxin	Solyc03g006360	+8.7	Auxin-repressed protein	Auxin repressed
	Solyc06g063060	+2.86	Auxin-repressed protein-like protein	Auxin repressed
	Solyc02g077880	+2.2	Auxin repressed/dormancy associated protein	Auxin repressed
	Solyc11g013310	−1.15	*LAX3*	Auxin influx carrier
	Solyc02g064830	−1.24	*GH3–3*	Auxin induced
	Solyc05g006220	−1.34	IAA-amino acid hydrolase ILR1 like	Auxin activation
	Solyc12g005310	−1.9	Auxin-responsive GH3 family protein	Auxin induced

a “+, increase; − reduction.

Gibberellins (GAs) and auxin play key roles in early fruit development. Among the 496 differentially expressed genes (DEGs), two genes (Solyc07g061730 and Solyc07g061720) encoding the GA-inactivating enzyme GA 2-oxidase were upregulated (log2FC = 2.09 and 1.24, respectively) and a key GA biosynthetic gene encoding GA20-oxidase-1 (Solyc03g006880) was downregulated (log2FC = −1.18) in L11 fruits (Table 2), suggesting that the concentrations of active GAs are reduced in CK-deficient fruits. In addition, a GA receptor gene (Solyc06g008870) was significantly upregulated in CK-deficient fruits (log2FC = 1.28; Table 2).

Seven auxin-related genes were significantly differentially expressed in CK-deficient fruits. Among them, three genes (Solyc03g006360, Solyc06g063060, and Solyc02g077880) encoding auxin-repressed proteins were significantly upregulated in CK-deficient fruits (log2FC = 8.7, 2.86, and 2.2, respectively). Four genes were downregulated in CK-deficient fruits—*LAX3* (Solyc11g013310, encoding an auxin influx carrier), two auxin early-induced *GH3* genes (Solyc02g064830 and Solyc12g005310), and indole-3-acetic acid (IAA)-amino acid hydrolase *ILR1* (Solyc05g006220; Table 2). These results suggest that auxin signaling is repressed in CK-deficient fruits.

To validate the RNA-Seq data, three upregulated and three downregulated genes in 5-DAA fruits of L11 and WT plants were subjected to qRT–PCR. The six genes showed the same differences in expression between L11 and WT fruits by both methods (Supplementary Fig. S3). The coefficient of regression between the two datasets was 0.9383 (Supplementary Fig. S3), suggesting that the RNA-Seq data are reliable.

### Effects of cytokinin deficiency on the concentrations of auxin and gibberellins

**Figure 5:**
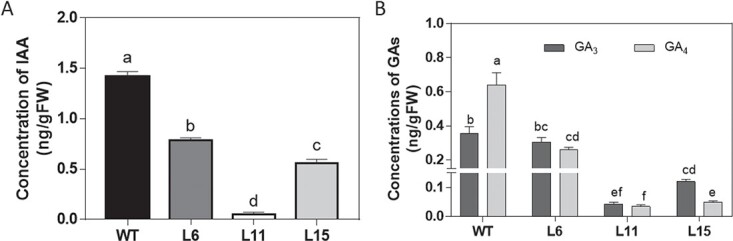
Effects of CK deficiency on concentrations of IAA (**a**) and GAs (**b**) in tomato fruits. Fruits at 5 days after anthesis were used to measure IAA and GA concentrations by UHPLC-TQ-MS/MS. Different letters indicate significant differences at the *P* < .05 level (*n* = 3).

As we expected, the concentrations of IAA and active GAs (GA_3_ and GA_4_) were decreased in 5 DAA fruits of these three transgenic lines. The level of IAA in the L11 fruits was only 3.7% of that in WT fruits (Fig. 5a). The concentrations of GA_3_ and GA_4_ were 12.3% and 5.5% of the level of WT control fruits, respectively (Fig. 5b).

## Discussion

### Endogenous cytokinin regulates fruit size and weight

CKs regulate cell division and possibly early fruit growth and development [3, 21]. However, no direct genetic evidence supports a role for endogenous CKs in the growth or development of specific fruit tissues. Using the fruit-specific promoter *Tmf7*, we overexpressed *AtCKX2* in tomato fruits. The expression level of the *AtCKX2* fusion gene was high in the early stages of transgenic fruit development. In addition, endogenous CK levels were reduced in *Tmf7::AtCKX2* fruits (Fig. 1), in agreement with reductions in the concentrations of CKs in *CKX*-overexpressing *Arabidopsis* [10], tobacco [22], oilseed rape [23], and poplar [12].

The reduction of CK levels in fruits leads to reduced fruit size and weight, seed number, and seed weight. The use of a fruit-specific gene promoter to control *CKX* expression can reduce the potential indirect effects of reductions in CK levels in leaf, stem, and root tissues on fruit growth and development. Using the *Tmf7* promoter to control *AtCKX2* expression, we showed that reduction of the CK levels in fruit tissues results in significant reductions in fruit size and weight. Such reductions were correlated with the magnitude of the CK level reductions in fruits of the transgenic lines L6, L15, and L11 (Fig. 2). Furthermore, exogenous application of CPPU to L11 fruits can increase single fruit weight and pericarp thickness (Supplementary Fig. S1). These results demonstrate that endogenous CKs play an important role in the regulation of fruit size and weight. CKs also regulate seed development in plants such as rice and barley [24–27]. The number of seeds per fruit and the weight of individual seeds were significantly reduced in CK-deficient transgenic fruits (Fig. 3), consistent with previous reports. However, the effects of reduced CK levels on seed growth and development were of lesser magnitude than were those on the pericarp and locular gel. One possible explanation is that the activity of the *Tmf7* promoter is less in developing seeds than in the pericarp and locular gel [16].

### Endogenous cytokinin regulates cell division and expansion in pericarp tissues

The reduction of CK concentrations in fruits reduced cell division in pericarp tissues. Fahima *et al*. [28] reported that exogenous application of CPPU, a synthetic cytokinin, to lichi fruits during the pericarp cell-division phase induced parenchymal and brachysclereid cell proliferation and resulted in thicker pericarps in mature fruits. Matsuo *et al.* [3] exogenously applied CPPU to unpollinated tomato ovaries, which induced parthenocarpy, mainly by promoting cell division. Moreover, during tomato fruit development, the cell division stage begins at anthesis and continues for ~2 weeks after fertilization, followed by a growth phase caused by cell expansion [29]. However, because the effects of the exogenous application of abnormally high doses of hormones do not necessarily reflect the roles of their endogenous counterparts, these results do not conclusively demonstrate that endogenous CKs play a role in fruit or pericarp growth. We have shown that the reduction of endogenous CK levels in fruit tissues leads to reductions in pericarp size and thickness. The reduced pericarp thickness was caused by decreased cell numbers and cell layers in CK-deficient transgenic fruits. Moreover, cell size was reduced in pericarp tissues of CK-deficient transgenic fruits (Fig. 4). The expression of *AtCKX2* was relatively high from 5 to 20 DAA (Fig. 1a), leading to reductions in endogenous CK levels in transgenic fruits. In sum, the results show that endogenous CKs play important roles in cell division and enlargement of pericarp tissues in the early stages of fruit development, and consequently determine pericarp thickness and fruit size.

Cyclins and cyclin-dependent protein kinases regulate cell division in tomato plants [17]. The D-type cyclin CYCD3 was reported to mediate CK-activated cell division in *Arabidopsis* [30, 31]. The expression of *CYCU1;1* (cyclin U1;1), *CYCU4;1*, and *CKI* was downregulated significantly in CK-deficient transgenic fruits (Table 1), suggesting that the effect of CKs on cell division in pericarp tissues is mediated by the activation of *CYCU1;1*, *CYCU4;1*, and *CKI*.

Our results also demonstrate that CKs play a role in cell expansion in tomato pericarp tissues. Cell enlargement depends largely on the cell turgor pressure, which is influenced by cell-wall loosening [32]. *PME* expression reportedly enhances cell-wall stiffening [33, 34]. In tomato, antisense-mediated suppression of a *PME* gene (*Pmeu1*) increased the rate of fruit softening during ripening [35]. The expression of two *PME* genes (Solyc07g064170 and Solyc01g091050) was significantly upregulated in CK-deficient transgenic fruits (Table 1), suggesting that *PME* expression is suppressed by CKs and is involved in the stiffening of tomato pericarp cell walls and reduction of pericarp cell expansion.

### Auxin and gibberellin are involved in cytokinin-regulated pericarp growth

Auxin and GA have been reported to mediate tomato fruit growth; increases in their contents and signaling accelerated fruit growth [36–38]. In this study, expression of the auxin influx carrier *LAX3* was repressed in CK-deficient fruits. Several auxin early-induced genes were downregulated and three auxin-repressed genes were upregulated in transgenic fruits (Table 2). Also, the concentration of IAA was reduced in the CKX transgenic fruits (Fig. 5a), suggesting that auxin is involved in the CK-regulated pericarp growth and development. Moreover, the expression of GA-related genes was altered in CK-deficient transgenic fruits. GA 2-oxidase genes, which are responsible for GA inactivation, were upregulated and GA 20-oxidase genes, which are responsible for GA biosynthesis, were downregulated, resulting in the reduction of active GA content in CK-deficient CKX-overexpressing transgenic fruits (Table 2; Fig. 5b). These results provide support for the idea that GAs are involved in CK-regulated pericarp growth and development.

A number of genes controlling fruit weight and size, including *EXCESSIVE NUMBER OF FLORAL ORGNAS* (*ENO*) [39], *WUSCHEL* (*WUS*) [40], *CLAVATA3* (*CLV3*) [41], *SUN* [42], *OVATE* [43], *KLUH* [44], and *CELL NUMBER REGULATOR* (*CNR*) [45], have been cloned. ENO, WUS and CLV3 are involved in the regulation of floral meristem activity, and consequently fruit size [39–41, 46]. These genes are not expressed or expressed at very low levels in 5 DAA fruits of WT and L11 plants (Supplementary Supplementary Table S2). In the regulation of shoot apical meristem development, auxin and CK signaling pathways are tightly connected to the WUS–CLV feedback loop [47]. Further investigation needs to be carried out to interpret the interactions of WUS and plant hormones in the regulation of fruit development. SUN and OVATE regulate cell number along different axes of the ovary and modulate fruit shape [42, 43, 46]. CNR is a negative regulator of cell division during fruit development [45]. *KLUH*, encoding a cytochrome P450, is involved in the regulation of fruit and seed weight [44]. In this study, we found that there were no significant differences in the expression of these genes between L11 and WT fruits at 5 DAA (Supplementary Table S2). These results indicated that these genes may act upstream of the CK signaling pathway or may not be involved in CK-regulated fruit development. The latter could mean that the CK-mediated regulation of fruit size may go through a slightly different pathway. On the other hand, for the *SUN* gene, which has been previously shown to be involved in the control of calcium-regulated processes [48], we also noticed that a calcium-binding protein PBP1-like gene (Solyc10g006700) and a calcium-dependent protein kinase gene (Solyc12g005030) were downregulated in the L11 fruits. Further, another *OVATE* family gene, *OFP20*, involved in fruit shape and size development [49, 50], was significantly down-regulated in L11 fruits at 5 DAA (Supplementary Table S2).

**Figure 6:**
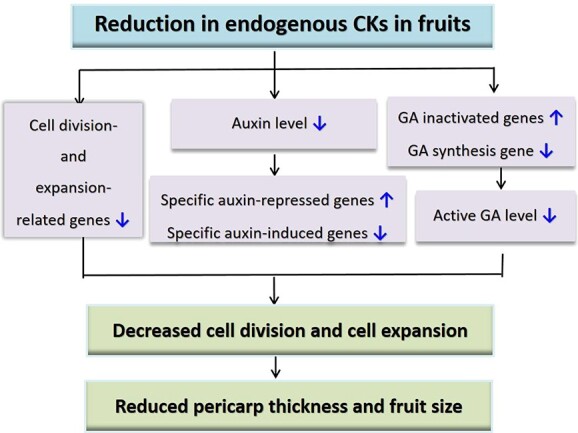
A working model for molecular mechanisms by which reduction in endogenous CKs reduces pericarp thickness and fruit size. ↑ represents increase and ↓ represents reduction.

In conclusion, the reduction of endogenous CK levels caused by overexpression of the CK catabolism gene *AtCKX2* in developing fruit tissues resulted in reductions in pericarp cell number and size and subsequently fruit size (Fig. 6). Lower levels of CKs lead to the downregulation of GA biosynthetic genes and upregulation of GA-inactivating genes, and therefore reductions in active GA levels. Auxin biosynthesis and signaling pathways also are repressed in CK-deficient pericarp tissues, as indicated by the downregulation of auxin-induced genes and upregulation of auxin-repressed genes. Changes in the expression of cell division- and expansion-related genes in CK-deficient transgenic fruits are responsible for the reduced pericarp thickness and fruit size.

## Materials and methods

### Plant materials and growth conditions

Tomato (*S. lycopersicum* ‘Micro-Tom’) and *AtCKX2*-overexpressing plants were used. Plants overexpressing *AtCKX2*, controlled by *Tmf7* (L6, L11, and L15), were produced in our laboratory. Tomato plants were grown in a greenhouse at 25°C with a 14-h light (intensity, 250 μmol m^−2^ s^−1^)/10-h dark cycle.

### Extraction and purification of cytokinins, auxin and gibberellins

CKs, IAA and GAs were extracted from samples of tomato fruits harvested from WT and transgenic plants at 5 DAA and purified according to the method of Niu *et al*. [51] with some modification. Briefly, 1 g (fresh weight) fruit tissues was ground to a powder in liquid nitrogen and placed in 10 ml 2-propanol/water/concentrated HCl (200:100:0.2, v/v/v). The samples were placed on a shaker at a speed of 100 rpm for 12 h at 4°C. Then, 15 ml dichloromethane was added and the mixture was incubated at 4°C for 1 h with shaking. After the solution was completely layered, the lower organic phase was passed through a 0.45 μm filter. The samples were dried and re-dissolved in 200 μl methanol and then 200 μl water. After centrifugation (10 min, 12 000 rpm, 4°C), the supernatant was passed through a 0.22 μm filter and then analyzed by UHPLC-TQ-MS/MS.

### UHPLC-TQ-MS/MS conditions

Hormones were analyzed on an Agilent 1290 UPLC system (Agilent, USA) coupled with an AB SCIEX QTRAP^®^ 6500 mass spectrometer (Applied Biosystems, USA). An Agilent Poroshell 120 SB-C18 column (2.1 mm × 150 mm, 2.7 μm) was used for separation at a flow rate of 0.3 ml/min. The hormone standard sample (Sigma-Aldrich) was dissolved in methanol. The mobile phase was composed of 0.1% formic acid in water (phase A) and methanol (phase B). A gradient program with the following proportions (v/v) of solvent A was applied: 0–1 min at 80%, 1–3 min from 80% to 50%, 3–9 min from 50% to 20% and held for 1.5 min, then returned to initial conditions. The injection volume was 1 μl. The electrospray ionization interface in the positive and negative multiple reaction monitoring mode was used for the analysis of hormones.

### Measurement of fruit size, weight, and pericarp thickness

Fruit length and diameter of fruits at the red stage were measured using Vernier calipers, and the weights of fruits at the red stage and parts of them were determined using a precision balance. The pericarp thickness of fruits was measured using Vernier calipers. At least 15 fruits per sample were analyzed.

### Histological analysis

Fruits were analyzed histologically at the mature green stage. Fruit tissues were fixed and embedded, and sections were made as described by Czerednik *et al*. [52]. The sections were stained with toluidine blue, and slides were scanned using an automated Pannoramic SCAN 150 device (3DHistech). The scanned slides were analyzed with Pannoramic Viewer software (3DHistech).

The number of pericarp cell layers was counted as described by Carrera *et al*. [53]. The average cell size was calculated by measuring the sizes of 30 medium-sized cells in the internal mesocarp in transverse sections. Measurements from three views per fruit of at least three fruits were obtained.

### Forchlorfenuron treatment

CPPU was purchased from Shanghai Yuanye Bio-Technology Co., Ltd (Shanghai, China). During flower anthesis, 0.1 mM CPPU was applied to the roots at the rate of 10 ml per plant every 3 days, for a total of five times. The control plants were treated with deionized water. For each treatment, we kept the flowers that bloomed that day and cut off the flowers that had opened. Fruits at the red stage were used to test single fruit weight and pericarp thickness.

### RNA-Seq and analysis

Total RNA was extracted from fruits of WT and L11 plants (three biological replicates each) at 5 DAA. The samples were sent to BMK Company (Beijing, China) for transcriptome sequencing using an Illumina HiSeq2500 platform. Clean data were obtained by removing low-quality reads, reads containing adapters, and reads containing poly-N from the raw data. These clean reads were mapped to the tomato genome reference (ITAG 3.2). Gene expression levels were estimated as FPKM. DEGs were identified using DEseq. The resulting *P* values were adjusted using the Benjamini and Hochberg approach to control the false discovery rate (FDR). Genes with FDRs <0.05 and |log2 (fold change)| ≥1 were classified as differentially expressed.

### RNA extraction and quantitative RT–PCR

Fruits at 5 DAA were ground into fine powder in liquid nitrogen and subjected to total RNA extraction and RT–qPCR [54]. Supplementary Table S3 shows the primer sequences used for RT–qPCR in this study.

## Acknowledgements

This work was supported by the National Natural Science Foundation of China (31672124 to L.G.).

## Author contributions

L.G. and Y.L. designed the experiments; M.S., X.W., H.L. and X.L. performed the experiments; M.S., X.L., L.G., and Y.L. analyzed the data; L.G. and Y.L. wrote the article.

## Data availability

The authors declare that the data supporting the study findings are presented in the article and additional supporting files are available from the corresponding author (L. G.) upon request.

## Conflict of interest

The authors declare no competing interests.

## Supplementary information

Supplementary data is available at *Horticulture Research Journal* online.

## Supplementary Material

Web_Material_uhab041
