# Supplementary material for: Cytokinins are involved in regulation of tomato pericarp thickness and fruit size
Source: Hortic Res. 2022 Jan 19;9:uhab041. doi: 10.1093/hr/uhab041 (PMC8968492; doi:10.1093/hr/uhab041)
Supplement: Web_Material_uhab041 [file web_material_uhab041.zip › Supplementary Tables and figures.docx]

**Supplementary Table 1 The quality of sequencing data in three replicates for the samples of wild type and transgenic L11**

| **Samples** | **Total Reads** | **Clean reads** | **Clean bases** | **%≥Q30** | **Uniquely Mapped Reads** |
| --- | --- | --- | --- | --- | --- |
| WT-1 | 48,660,934 | 24,330,467 | 7,255,651,560 | 95.49% | 46,139,347 |
| WT-2 | 45,934,102 | 22,967,051 | 6,859,833,940 | 95.32% | 43,627,163 |
| WT-3 | 50,174,656 | 25,087,328 | 7,489,295,312 | 95.29% | 47,587,827 |
| L11-1 | 44,152,224 | 22,076,112 | 6,588,442,720 | 95.09% | 41,791,093 |
| L11-2 | 50,086,702 | 25,043,351 | 7,485,942,958 | 94.97% | 47,468,838 |
| L11-3 | 45,374,038 | 22,687,019 | 6,781,223,154 | 95.39% | 42,750,121 |

| Supplementary Table 2 Expression levels of fruit size-related genes in L11 fruits relative to those in WT fruits | | | | | | | | | |
| --- | --- | --- | --- | --- | --- | --- | --- | --- | --- |
|  |  |  |  |  |  |  |  |  |  |
| **Gene ID** | **Gene**  **name** | **WT-1**  **FPKM** | **WT-2**  **FPKM** | **WT-3**  **FPKM** | **L11-1**  **FPKM** | **L11-2 FPKM** | **L11-3 FPKM** | **log2FC** | **Regulated** |
| Solyc03g117230 | ENO | 0.000 | 0.000 | 0.000 | 0.000 | 0.000 | 0.000 | -- | -- |
| Solyc02g083950 | WUS/LC | 0.028 | 0.000 | 0.000 | 0.062 | 0.041 | 0.015 | -- | -- |
| Solyc11g071380 | CLV3 | 0.187 | 0.000 | 0.000 | 0.000 | 0.000 | 0.000 | -- | -- |
| Solyc10g079240 | SUN | 0.776 | 1.068 | 0.755 | 0.912 | 0.602 | 0.516 | -0.359 | normal |
| Solyc02g085500 | OVATE | 5.635 | 5.375 | 4.353 | 5.232 | 2.907 | 4.066 | -0.334 | normal |
| Solyc03g114940 | KLUH | 86.066 | 89.138 | 87.235 | 91.652 | 91.030 | 85.519 | 0.030 | normal |
| Solyc05g051690 | CNR | 13.111 | 11.727 | 13.089 | 13.817 | 11.731 | 13.014 | 0.023 | normal |
| Solyc10g006700 | calcium-binding protein PBP1 | 15.929 | 25.992 | 19.340 | 7.184 | 8.614 | 10.641 | -1.209 | down |
| Solyc12g005030 | calcium-dependent protein kinase | 19.936 | 39.167 | 35.775 | 7.384 | 10.057 | 9.121 | -1.850 | down |
| Solyc10g076180 | OFP20 | 13.001 | 13.715 | 14.075 | 3.834 | 3.820 | 4.108 | -1.792 | down |

**Supplementary Table 3 PCR Primers used in this study**

| **Gene Name/ID** | **Forward primer** | **Reward primer** |
| --- | --- | --- |
| AtCKX2 | TTACTTGCCCGGGTTCATATC | GTAGAGGTTAAGCCAAGGATGAG |
| Solyc03g019820 | CACCTTGTCTGAGCTCTTATCC | CGCCAACGCTATCACTATCA |
| Solyc03g097870 | GTTGGTGGATTTGGTGCAATAG | TGCCTAAGGGAGCAACAAATA |
| Solyc12g098900 | GGACAATACGTAGCAGGGAATC | GCAGTCTCGTATGCTCTGTTT |
| Solyc07g061750 | GCCAGAGCTAAGAGAGAGAAAC | ACCTCCTGGTAATGTGAAACC |
| Solyc06g017860 | GAATGGTCTGGGCAGAAAGA | CATCATGGACCCTAAGGAATGT |
| Solyc06g035940 | GCTCCCTTCTGGTTGCATTA | GCTCTGATTAAAGGGCGATACA |
| Actin | TGTCCCTATTTACGAGGGTTATGC | CAGTTAAATCACGACCAGCAAGAT |

**Supplementary Figure 1**

**
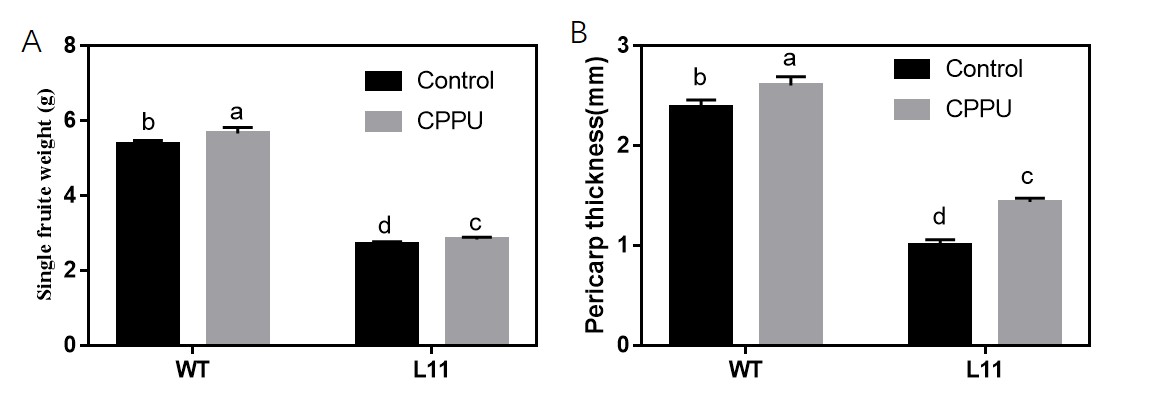
**

Supplementary Figure 1 Effects of CPPU on single fruit weight (A) and pericarp thickness (B)

in mature red fruits.

During flower anthesis, 0.1 mM CPPU was applied to the roots at the rate of 10 ml per plant every 3 days, totally 5 times. Different letters indicate significant differences at the *P* < 0.05 level (n=15).

**Supplementary Figure 2**


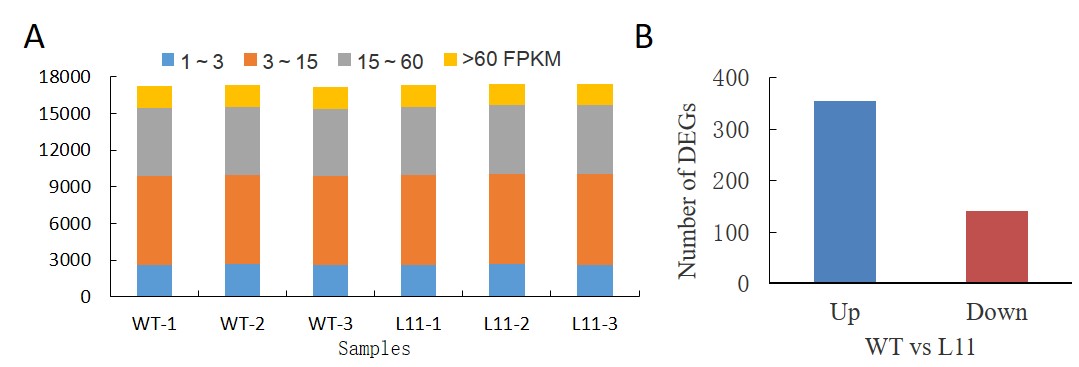


**Supplementary Figure 2 Analysis of gene expression in fruits from wild type and transgenic L11.**

(A) Number of genes expressed in each sample with FPKM value higher than 1;(B) Number of DEGs between wild type and transgenic L11.**Supplementary Figure 3**

**
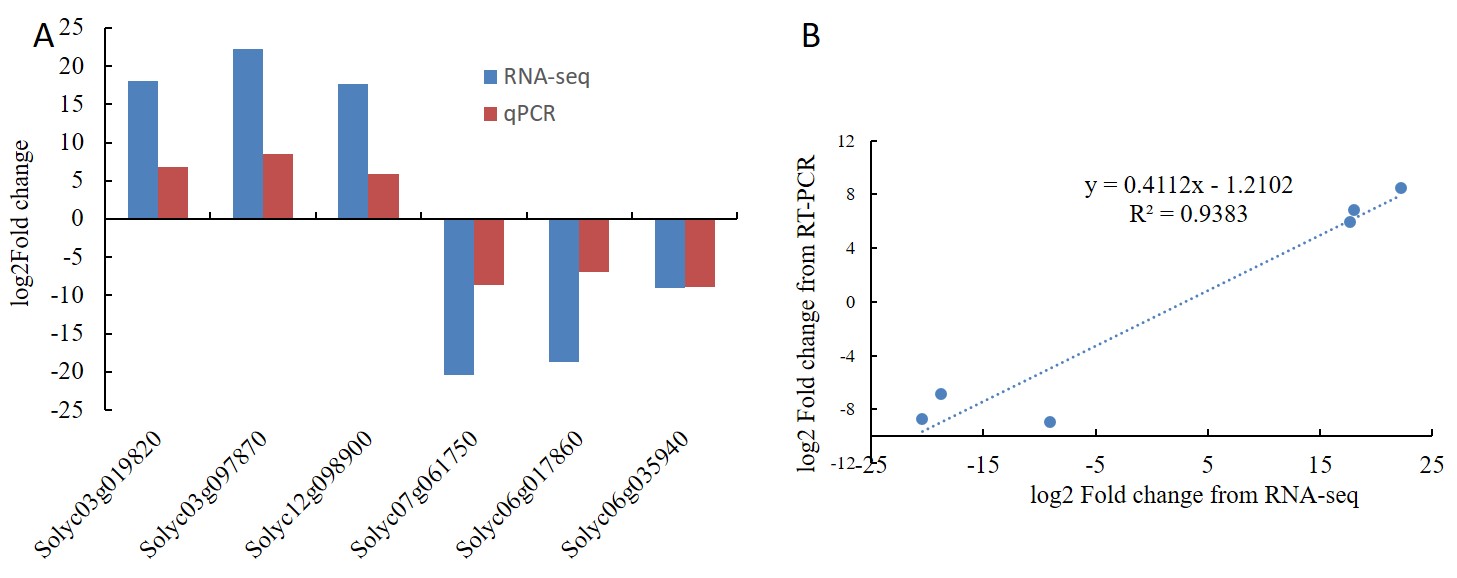
**

**Supplementary Figure 3 Validation of RNA-seq data by qRT-PCR.**

(A) Fold changes of transcript levels from 3 up-regulated genes and 3 down-regulated genes in 5 DAA fruits of L11 as compared to thoes in WT plants measured by RNA-seq and qRT-PCR; (B) Linear regression analysis for the fold changes among 6 genes for RNA-seq data and qRT-PCR data.
